# A digital-twin platform for cryospheric disaster warning

**DOI:** 10.1093/nsr/nwae300

**Published:** 2024-08-24

**Authors:** Yifei Cui, Yao Li, Hui Tang, Jens M Turowski, Yan Yan, Nazir Ahmed Bazai, Ruilong Wei, Li Li

**Affiliations:** State Key Laboratory of Hydroscience and Engineering, Department of Hydraulic Engineering, Tsinghua University, China; Key Laboratory of Hydrosphere Sciences of the Ministry of Water Resources, Tsinghua University, China; State Key Laboratory of Hydroscience and Engineering, Department of Hydraulic Engineering, Tsinghua University, China; Section 4.7: Earth Surface Process Modelling, GFZ German Research Centre for Geosciences, Germany; Section 4.7: Earth Surface Process Modelling, GFZ German Research Centre for Geosciences, Germany; Section 4.6: Geomorphology, GFZ German Research Centre for Geosciences, Germany; Key Laboratory of High-Speed Railway Engineering, MOE/School of Civil Engineering, Southwest Jiaotong University, China; China Pakistan Joint Research Centre on Earth Sciences HEC-CAS, Pakistan; Key Laboratory of Mountain Hazards and Surface Process, Institute of Mountain Hazards and Environment, CAS, China; Institute of Geophysics, China Earthquake Administration, China

## Abstract

With increasing glacial hazards due to global climate warming, a promising digital-twin-based platform has been proposed for early warning of cryospheric disasters.

At present, human society all around the world is being challenged by the unprecedented crisis of global warming, which has caused dramatic changes in weather patterns and extremes. The rising temperature has rapidly evolving consequences especially in glacierized environments. Excluding the large Antarctic and Greenland ice sheets, there are nearly 200 000 glaciers with an area of 726 000 km^2^ all over the world [1]. During 2000–2019, these glaciers lost 267 ± 16 Gt yr^−1^ in mass due to climatic warming [2]. Since 1990, shrinking and receding glaciers have not only led to an increase in the volume of global glacier lakes by ∼48%, but also deposited large amounts of sediment [1]. Such a rapid cryosphere change has enhanced the risk of cryospheric disasters such as glacial lake outburst floods (GLOF), ice-rock avalanches and glacial debris flows, posing severe threats to downstream communities (Fig. 1) [3,4].

**Figure 1.**
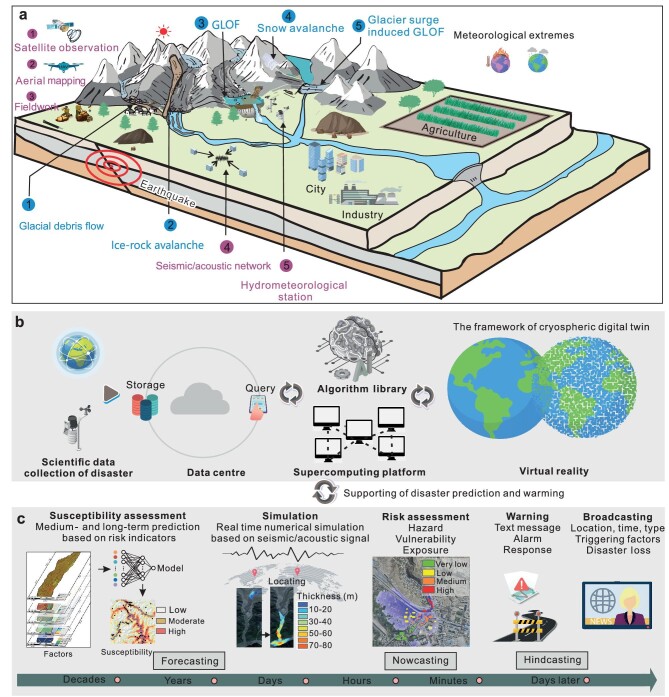
Conceptualized framework of a digital-twin-based early-warning system for cryospheric disasters. (a) Various hazard types and their scientific data collection in the cryospheric environment. There are five kinds of cryospheric disasters (indicated by blue text) resulting from glacier instability. Such events can cause serious damage to local infrastructures and pose a threat to the water security of downstream communities. Five technical methods (indicated by purple text) can be used to inform the formation, evolution and early warning of cryospheric disasters. The integration of multisource data and their associated inversion algorithms presents challenges in the development of a comprehensive disaster early-warning platform. (b) The digital twin is constructed using virtual reality, supercomputing and cloud sharing technology. (c) Prediction, risk management and early warning at different stages of disaster events. Virtual reality in a digital twin provides an interactive window for requirement uploading and result display. With this window, the digital twin could guide the hazards as well as early warning and mitigation measures for the natural twin.

During the last decade, the community has mainly focused on understanding changes in catastrophic cryospheric disasters and their potential impacts [5,6]. However, there is limited capacity to respond to and mitigate such events, particularly in developing countries such as Pakistan, India and China. A key element for disaster mitigation is a robust and affordable early-warning system that delivers meaningful alerts to at-risk downstream communities. Such systems could support mitigation efforts and inform top-level policy planning and decision-making. A digital-twin (DT) platform could create a virtual model of a physical system, allowing real-time monitoring, simulation and analysis of disasters with high levels of physical and spatial accuracy [7]. In this perspective, we summarize the research objectives of cryospheric disaster early warning and introduce DTs as a potential solution to address these challenges.

## CHALLENGES IN EARLY WARNING OF CRYOSPHERIC DISASTERS

Cryospheric disasters in high-mountain environments present a complex interplay between different Earth surface processes. Various initiation mechanisms, cross-temporal scales, dynamic processes with feedbacks on multiple scales and challenges in setting up and maintaining monitoring networks make it difficult to observe and predict them. In the past decades, remote sensing, *in situ* observation, numerical simulations and burgeoning seismo-acoustic monitoring have been mainstream elements in the construction of early-warning systems [8,9]. These diverse techniques and their information retrieval methods are routinely used to assess disaster hazard and risk, predict the future occurrence of events (forecasting), provide a warning signal for approaching hazards (nowcasting) and analyse past events (hindcasting), respectively. However, these traditional disaster early-warning systems have the following shortcomings: (i) limited real-time data integration, leading to delays in data processing and interpretation; (ii) reliance on models calibrated on historical data without the capability themselves, making it difficult to update real-time changes in disaster development environments; (iii) a low degree of automation, relying on manual monitoring and response; (iv) limited integration between different data sources and systems.

A robust early-warning system should take into account the entire process of disaster development. First, before an event occurs, historical records and data on topography, geology, meteorology and surface deformation from satellite images and *in situ* monitoring should be analysed to identify its controlling factors, triggering thresholds and evolution processes. Second, susceptibility and risk assessment of cryospheric disasters should be carried out to identify glacial catchments at high risk as a long-term observation target [10]. Third, integrating hypothetical simulations of different types of cryospheric disasters (such as GLOF, ice-rock avalanche and glacial debris flow) in the target region with its real-time observations could produce timely warning information and help evaluate impending damage. The real-time observations can include seismo-acoustic signals detected by using seismometers and geophones, hydro-meteorologic data [11], remote-sensing observations [12] and detailed mapping and analysis of landscapes and climate in and beyond the targeted region at high risk [13].

To construct a system with the outlined capabilities, there are four challenges to address: (i) realizing multisource sensor interconnection and data management; (ii) integrating information extraction with a library of algorithms for analysis and prediction based on structured and unstructured data (e.g. imaging, text and binary data); (iii) possessing rapid numerical computing capability of events for real-time damage assessment; and (iv) constructing a visualized and complete workflow including prediction, rapid simulation, quantitative risk assessment, early-warning decision-making and early-warning release.

## A PROMISING DT SOLUTION

Advanced early-warning systems are vital to accurately predict and mitigate cryospheric disasters and these must integrate the above-mentioned challenges such as data collection and management and event information extraction data with physics-based modeling. Building a DT platform is a ground-breaking solution offering digital replicas to simulate and monitor cryosphere changes (Fig. 1b). Specifically, the DT platform could allow scientists and decision-makers to analyse, visualize, monitor and forecast the impact of climate change and human activities on cryospheric changes, thereby revolutionizing the prediction and mitigation of cryospheric disasters.

Such a DT differs from existing early-warning systems in the following three aspects. First, it incorporates data, analysis and predictions across multiple timescales, and can utilize them by operating in different modes. In phases without cryospheric disaster events, it distinguishes hindcasting, in which available data are analysed to update thresholds and hazard scenarios, from forecasting and risk assessment, in which the data are used to predict the likelihood of events and their potential impacts. Typically, hazard and risk assessment produce probabilistic predictions, such as susceptibility maps, while forecasting provides estimates of the likelihood that an event will occur in the near future. In addition, the DT platform can support real-time reactions and mitigation efforts when an event ensues by supplying nowcasting predictions. With high-performance computing, the overarching objective is to provide reliable information promptly. This could involve real-time analysis of meteorological, seismo-acoustic or hydrometric data, as well as deterministic simulations using process-based models to identify impacted areas and inform rescue efforts. Second, the DT integrates a wide array of data sources covering different spatial and temporal scales, and can process a large volume of data. For example, remote-sensing data are typically available at coarse temporal resolution but cover large areas, while seismo-acoustic and hydro-meteorological data are recorded at a point with high temporal resolution. Third, the DT uses data science methods to automatically update its structure and interact with the physical twin, using incoming data and results from hazard and risk assessment, hindcasting and forecasting. For example, this two-way coupling system could use scenario simulation to guide the design and implementation of real-time monitoring networks and the construction of protective infrastructures in the natural twin, which are subsequently updated in the DT. During events, designed mitigation measures, such as the deployment of mobile flood fences, the opening of reservoir gates to control flood discharge and the evacuation of residents, feed back into the DT for an effective assessment and real-time adjustment when operating in the nowcasting mode. A DT early-warning system could therefore deal with all aspects of cryospheric disasters, including the support of hazard preparedness for threatened communities through hindcasting and forecasting, the provision of warnings in case of an event and the support of reactions and mitigation through nowcasting (Fig. 1c).

## VISION AND FUTURE WORKS

To build a DT for the early warning of cryospheric disasters, four key aspects need to be addressed in future work. First, an open, extensible and modular software framework and architecture are necessary. Traditional monitoring and early-warning systems usually rely on specific software development, limiting their ability to provide prediction and warning information for the entire evolution process of cryospheric disaster events [6]. The envisioned framework allows multi-structured data management and the integration of different algorithms for information extraction, analysis, forward modeling and inversion. In addition, the uniform system interface could be made accessible to all relevant researchers, users or contributors. The interface could also provide access to existing geoscience databases, such as EARTHDATA of United States Geological Survey. Second, there is a need for a library of algorithms for robust disaster identification that leverages all available data. While automatic detection algorithms using remote-sensing data are effective in low-altitude areas and vegetation-covered mountains, their performance is limited in bare alpine areas [14]. For cryospheric disaster detection, we recommend using advanced deep learning to process satellite images. This approach has worked well when extracting weak features and for change detection [15]. Meanwhile, disaster identification algorithms based on real-time seismo-acoustic monitoring data should also be integrated into the algorithm library [16]. The development of rapid numerical simulation models for real-time event simulation, as well as accurate machine-learning algorithms and more detailed physics-based models for creating a scenario database of potential events, is the third aspect that needs to be improved in the future. Parameters in numerical simulations of hazard processes are generally estimated from or calibrated based on prior events, which reduces the accuracy of real-time disaster prediction. A DT integrates real-time observation data and numerical simulations to provide dynamic model parameters or scenario selection from hindcasting and event analysis, and recalibrates as new parameters (type, flow properties, volume) become available during events [17]. In addition, to improve the robustness of scenario matching, the algorithms must be capable of updating themselves via parameter retraining as the observation data expand. Transformed deep-learning algorithms could be a potential option to meet this kind of challenge. The fourth and last aspect is to construct an optimal multisensor monitoring network. A dense and affordable real-time seismological and meteorological network is the key to a successful early-warning system using the DT approach [18]. Currently, in remote high-mountain areas, such as high-mountain Asia, Andes and Caucasus, the few available seismo-acoustic, meteorological and hydrological stations cannot provide sufficient data. Furthermore, existing instruments are rarely installed at optimal locations. Here, the DT approach could help in the design of optimal instrument networks.

Once the DT early-warning system has been developed, its implementation will require comprehensive promotion with a range of stakeholders. First, a unified data-sharing strategy, including cross-border and cross-organization cooperation and unified data standards, needs to be developed and agreed upon. Second, the collection and real-time transmission of monitoring data in the harsh cryospheric environment present another challenge that can be solved using advanced satellite communication technology. Finally, experts on the use of the DT, its maintenance and application, and ground-based sensor networks, as well as hazard mitigation in case of an event, need to be trained within local communities.

In conclusion, a DT platform represents a ground-breaking approach toward developing reliable early-warning systems for cryosphere hazards, encompassing the entirety of current approaches to preparation for cryospheric disasters. For example, the analysis of patterns and susceptibility utilizes research in physical geography, geographic information systems and remote sensing. The integration of expertise from fluid mechanics, granular flows, seismology and computational science is crucial for understanding disaster processes, constructing numerical models and performing parameter inversion from data streams. Furthermore, the development of the system demands close collaboration between software developers and the various research communities. Therefore, achievement of this work will require extensive interdisciplinary, transdisciplinary and international collaborations within the Earth science community.
